# Widespread Occurrence of *Batrachochytrium dendrobatidis* in Contemporary and Historical Samples of the Endangered *Bombina pachypus* along the Italian Peninsula

**DOI:** 10.1371/journal.pone.0063349

**Published:** 2013-05-07

**Authors:** Daniele Canestrelli, Mauro Zampiglia, Giuseppe Nascetti

**Affiliations:** Dipartimento di Scienze Ecologiche e Biologiche, Università della Tuscia, Viterbo, Italy; Leibniz Institute for Natural Products Research and Infection Biology- Hans Knoell Institute, Germany

## Abstract

*Batrachochytrium dendrobatidis* is considered a main driver of the worldwide declines and extinctions of amphibian populations. Nonetheless, fundamental questions about its epidemiology, including whether it acts mainly as a “lone killer” or in conjunction with other factors, remain largely open. In this paper we analysed contemporary and historical samples of the endangered Apennine yellow-bellied toad (*Bombina pachypus*) along the Italian peninsula, in order to assess the presence of the pathogen and its spreading dynamics. Once common throughout its range, *B. pachypus* started to decline after the mid-1990s in the northern and central regions, whereas no declines have been observed so far in the southern region. We show that *Batrachochytrium dendrobatidis* is currently widespread along the entire peninsula, and that this was already so at least as early as the late 1970s, that is, well before the beginning of the observed declines. This temporal mismatch between pathogen occurrence and host decline, as well as the spatial pattern of the declines, suggests that the pathogen has not acted as a “lone killer”, but in conjunction with other factors. Among the potentially interacting factors, we identified two as the most probable, genetic diversity of host populations and recent climate changes. We discuss the plausibility of this scenario and its implications on the conservation of *B. pachypus* populations.

## Introduction

Chytridiomycosis is an emerging infectious disease threatening amphibian populations and several authors have suggested that it is a primary driver of the worldwide amphibian declines [1]–[5]. The chytrid fungus *Batrachochytrium dendrobatidis* is the causative agent of this disease [6] and has been intensively studied in recent years (reviewed in [7], [8]). Nevertheless, many aspects of its epidemiology still remain unresolved, including its area of origin and spread dynamics [9]–[12].

Two main hypotheses have been proposed to explain chytridiomycosis outbreaks and disease dynamics [13]. The first hypothesis, called ‘novel pathogen hypothesis’, suggests a recent diffusion of the pathogen into novel geographic areas, where it has encountered ‘naïve’ hosts susceptible to infection [14], [15]. Under this scenario, a crucial causative agent of the disease spread would have been the human-driven introduction of infected individuals belonging to reservoir species [16]. The second hypothesis, called ‘endemic pathogen hypothesis’, suggests that recent outbreaks of *B. dendrobatidis* were driven by recent environmental changes, which would have affected the virulence of an endemic pathogen, host susceptibility or a combination of such factors [13]. Finally, since these two hypotheses are not mutually exclusive, a third scenario can be suggested, i.e. that the pathogen spread (either natural or human-driven) and various environmental changes could act as interacting factors in favouring disease outbreaks [17]–[19].

In the last decades, the first two hypotheses have been intensively explored (e.g., [13], [20], [21]). To disentangle among them, most studies have focused on the identification of possible patterns of wave-like die-offs of amphibian populations ([21]–[23] but see [24]), genetic imprints of a recent demographic/range expansion of the pathogen [25]–[28], and correlations between chytridiomycosis outbreaks and climate-related environmental factors [29]–[32]. Nevertheless, and in spite of the significant achievements in these fields, whether a single or multiple causes can account for the worldwide emergence of chytrid outbreaks remains disputed [33]. In light of this, growing emphasis has been recently placed on the comparative analysis of historical (i.e. pre-decline) vs. contemporary samples (e.g., [9], [34]–[36]). Indeed, evidence of the chytrid’s presence for a number of years in pre-decline (apparently healthy) amphibian populations would suggest that this fungus did not act as a ‘lone killer’ (contrary to the ‘novel pathogen’ hypothesis), but would support a role for other ‘conspiring’ factors in promoting declines, regardless of whether the pathogen is native or introduced.

Some of the first evidence for the occurrence of *B. dendrobatidis* in a declining amphibian in Europe concerned the Apennine yellow-bellied toad *Bombina pachypus* [37], a species endemic to the Italian peninsula. Once abundant throughout its range (it was categorized as ‘Least Concern’ in the IUCN Red List of Threatened Species until 2009 [38]), *B. pachypus* populations have been reported to decline in most parts of its natural range, with the exception of the southernmost portion (the Calabria region), since about the last 15 years [38], [39]. For instance, during a survey carried out in the years 1999–2000 [39], 82% of known sites of presence were confirmed within the Calabria region, whereas 54% were confirmed throughout the rest of the peninsula. Moreover, 64 new sites of presence were discovered in Calabria since 1996, while just 5 new sites were discovered in the other regions [39]. Consequently, the species is now considered as ‘Endangered’ by the IUCN [38], mostly owing to its susceptibility to chytridiomycosis. It is listed in the Annexes II and IV of the EU Habitat Directive, in the Appendix II of the Bern Convention and is protected by several national and regional laws in Italy. Nevertheless, no studies have been carried out to date in order to assess the distribution of *B. dendrobatidis* among populations neither of this species nor of other co-distributed amphibians.

In this work, using the recently described nested-PCR procedure [11], we carried out diagnostic tests for the occurrence of *B. dendrobatidis* on individuals of *B. pachypus* sampled throughout its range. Our aims were: 1) to assess the current distribution of *B. dendrobatidis* along the Italian peninsula and 2) to contribute to the above mentioned debate about disease dynamics by assessing whether *B. dendrobatidis* was already present in the area before the beginning of the observed decline or if its spread in the area could be reliably assumed to have occurred close to the time of the observed declines. With this aim, we analyzed individuals sampled recently (2003–2012) and earlier (1978–1981), in both cases spanning the range of the study species along the Italian peninsula.

## Materials and Methods

### Ethics Statement

*Bombina pachypus* individuals sampled recently were captured under a permit from the Italian Ministry of Environment (Direzione Generale per la Protezione della Natura). No permits were needed for sampling activities at the time of the first sampling session (1978–1981). We collected tissue samples from toe-tips, after anaesthetizing toads by submerging them in a 0.02% solution of MS222 (3-aminobenzoic acid ethyl ester). After the completion of the sampling procedure, the individuals were released at the collection site. Tissue samples were stored in 96% ethanol until further analyses.

### Sampling and Laboratory Procedures

**Figure 1:**
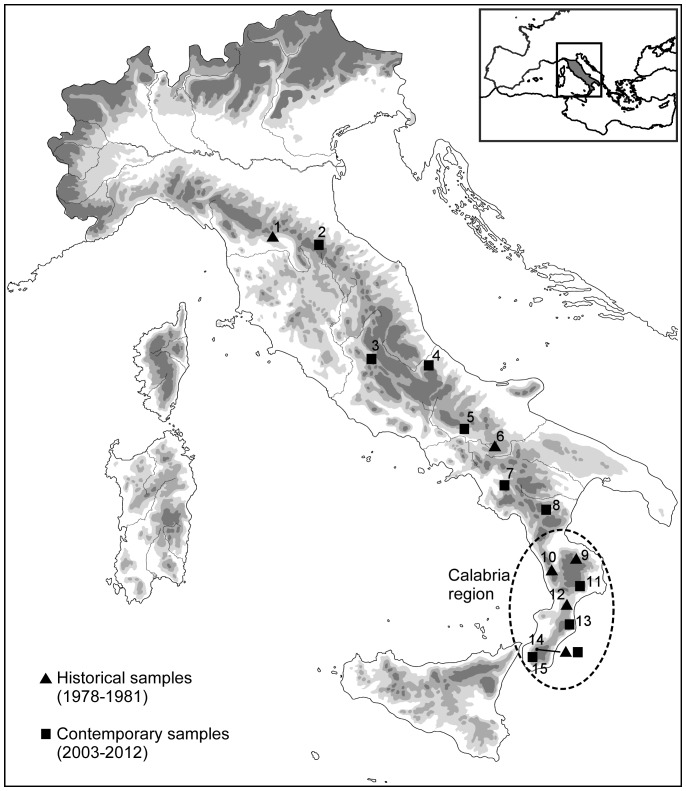
Geographic distribution of the 15 sampled populations of *Bombina pachypus*. The inset shows the species range along the Italian peninsula.

We analysed 136 individuals of *B. pachypus* from 15 sampling sites (17 samples, as two sites were sampled in two different years), 8 in the northern and central portion of the peninsula (sites 1 to 8; 82 individuals overall) and 7 from the Calabria region (southern Italy; sites 9 to 15; 54 individuals overall). Sampling sessions were carried out from late April to mid June. Sampling locations - spanning the entire Apennine peninsula -, as well as the number of individuals sampled at each site and year are presented in Table 1 and Figure 1. Among the tested individuals, 56 were collected during a sampling campaign carried out between 1978 and 1981 (38 were from Calabria and 18 were from populations located more to the north), while the remaining 80 samples were drawn between 2003 and 2012 (16 were from Calabria and 64 were from populations located more to the north).

**Table 1:** Geographic location and sampling year of the 15 populations of *Bombina pachypus* analysed, number of individuals analysed (n), and number of individuals testing positive for the presence of Batrachochytrium dendrobatidis (p).

	Site	LatitudeN	LongitudeE	Year	n	p
**1**	Rio a Buti	43° 56′	11° 6′	1978	13	9
**2**	Bagno diRomagna	43° 51′	11° 57′	2004	13	4
				2012	9	6
**3**	Stipes	42° 15′	12° 58′	2003	5	5
**4**	RoccaCaramanico	42° 09′	14° 01′	2003	6	3
**5**	San Lupo	41° 16′	14° 39′	2003	10	6
**6**	Carife	41° 01′	15° 12′	1981	5	2
**7**	S. Angeloa Fasanella	40° 27′	15° 20′	2003	19	15
**8**	S. SeverinoLucano	40° 05′	16° 04′	2003	2	2
**9**	MacchiaLonga	39° 22′	16° 36′	1981	5	2
**10**	MonteCocuzzo	39° 14′	16° 10′	1981	8	1
**11**	Taverna	39° 01′	16° 35′	2003	2	2
**12**	Cardinale	38° 44′	16° 24′	1978	15	7
**13**	Stilo	38° 30′	16° 25′	2003	11	10
**14**	Gambarie	38° 10′	15° 50′	1981	10	8
				2003	2	2
**15**	Cardeto	38° 05′	15° 46′	2006	1	1

Genomic DNA was extracted from single toes stored at −80°C, following the protocol by Boyle et al. [40] with some modifications. Skin swabbing, although more sensitive than toe-clipping as sampling method [41], was not used for this study since whole bodies were not available for the historical samples. We added 50 µL of PrepMan Ultra (Applied Biosystems) to each tissue sample, with 30–40 mg of glass beads measuring 0.4–0.6 mm diameter (Sartorius). Samples were homogenized for 1 min at 3000 rpm in a Mikro-Dismembrator S (Sartorius) and centrifuged for 30 sec at 13000× *g* in a microfuge (Eppendorf Centrifuge 5415 D). The homogenization and centrifugation procedure was repeated twice. Samples were then boiled for 10 min, cooled at room temperature for 2 min, and centrifuged at 13000× *g* for 3 min. Next, 20 µL of supernatant were retained and stored at −80°C until further analysis.

In order to assess the presence/absence of *B. dendrobatidis* DNA within the extracted DNA, we used the nested-PCR protocol recently developed by Goka et al. [11]. This method has been shown to increase specificity and sensitivity by a factor of 10 and 100 (see [11]), compared to real-time TaqMan PCR assays [40] and single round PCR assays respectively [42]. The target DNA was amplified twice using 2 different pairs of primers. The first PCR was performed using the primer pair Bd18SF1 (5′-TTTGTACACACCGCCCGTCGC-3′) and Bd28SR1 (5′-ATATGCTTAAGTTCAGCGGG-3′). The reaction mix (25 µL) contained: 5 µL of template DNA diluted 1∶10 with distilled water, 0.5 µM of each primer, 2 mM MgCl_2_, 0.2 mM of each dNTP, Colorless GoTaq Reaction Buffer 1× and 1 U of GoTaq Polymerase (Promega). The PCR cycling conditions were as follows: an initial denaturation step for 9 min at 95°C, 30 cycles of 30 sec at 94°C, 30 sec at 50°C and 2 min at 72°C and a final extension step of 7 min at 72°C [11]. The second PCR was performed using the primer pair Bd1a (5′CAGTGTGCCATATGTCACG-3′) and Bd2a (5′- CATGGTTCATATCTGTCCAG-3′) [42]. The reaction mix (25 µL) contained: 2 µL of the first PCR product used as template, 0.5 µM of each primer, 2 mM MgCl_2_, 0.2 mM of each dNTP, Colorless GoTaq Reaction Buffer 1× and 1 U of GoTaq Polymerase (Promega). The PCR cycling was as follows: an initial denaturation for 9 min at 95°C, 30 cycles of 30 sec at 94°C, 30 sec at 65°C and 30 sec at 72°C and a final extension step of 7 min at 72°C [11].

Each PCR assay was performed in duplicate, and we considered the test positive when PCR products of the expected size (approximately 300 bp) were observed at least once. We included in each assay a positive control with DNA extracted from *B. dendrobatidis* zoospores (JEL423 kindly provided by Prof. Joyce Longcore, University of Maine) and a negative control of DNA-free distilled water. PCR products (if any) were separated and visualized on a 1% agarose gel. To confirm that the amplification products were from the *B. dendrobatidis* genome, a randomly selected 13% of the PCR products were double-sequenced (n = 11) and compared with reference sequences in GenBank. Sequences were carried out by Macrogen inc. (www.macrogen.com) using an ABI3730XL.

## Results

Nested PCR diagnostic tests for *B. dendrobatidis* presence/absence yielded positive results in 85 out of 136 individuals analysed (62.5%). In all cases the diagnostic PCR bands were of the expected size (approximately 300 bp; see [11], [42]). Among the 11 PCR products that were sequenced, percent identity with *B. dendrobatidis* sequences available in GenBank was always 100%, and in no case organisms other than *B. dendrobatidis* were among the first 100 blast hits.

The geographic and temporal distributions of the individuals tested positive are shown in Table 1 and Fig.1. *B. dendrobatidis* positives were found in all sampled populations, including the oldest samples analysed (sample 1 and 12; year 1978). Moreover, the prevalence of *B. dendrobatidis* positivity was never below 12%, with frequencies ≥70% observed in most samples (samples 3, 7, 8, 11, 13, 14 and 15).

Prevalence of *B. dendrobatidis* significantly increased over time. Indeed, 51.8% of individuals analyzed in 1978–1981 tested positive, compared to 70% in 2003–2012 (Fisher exact test, P = 0.0472). Nevertheless, this pattern was not apparent throughout the species range. For the Calabrian localities (9–15), the difference between the two periods is striking: 47.4% in 1978–1981, compared to 93.8% in 2003–2012 (Fisher exact test, P = 0.0017). However, for the northern localities (1–8), overall prevalence of the chytrid did not differ significantly between the two periods: 61.1% in 1978–1981 versus 64.1% in 2003–2012 (Fisher exact test, P = 1.00).

Finally, differences in *B. dendrobatidis* prevalence among northern (i.e. declining) and Calabrian (i.e. non-declining) populations appeared to have accumulated over the study period. Indeed, in the early sampling (1978–1981), there appears to be no effect of latitude: 61.1% in the north versus 47.4% in Calabria (Fisher exact test, P = 0.3990). However, in the recent sampling (2003–2012) there is a significant difference between northern and Calabrian populations: 64.1% vs. 93.8% respectively (Fisher exact test, P = 0.0300).

## Discussion

Since the first evidence for the occurrence of *B. dendrobatidis* infections in an amphibian species in Italy [37], several investigations have been carried out to assess its presence/absence at specific locations, mostly in northern Italy [16], [43]–[45]. This is the first study carried out using range-wide sampling, spanning the entire peninsula and using both historical and contemporary samples. Our results clearly showed that *B. dendrobatidis* has a widespread distribution along the Italian peninsula, and that it was already present throughout this area, at least since 1978.

Among the studied populations of *B. pachypus*, the occurrence of *B. dendrobatidis* was conspicuous: it was found in all the sampled populations and with high overall infection prevalence (62.5% of the individuals analysed). Moreover, chytrid prevalence could be even higher than suggested by our data (due to the likelihood of false negatives associated with the toe-clipping method; [41]). When combined with previous observations of chytrid-driven mortality in captive individuals, a role for *B. dendrobatidis* in the widespread decline of *B. pachypus* appears especially plausible [37]. Nevertheless, at least two main issues emerging from our data indicate that the pathogen’s distribution alone cannot explain the patterns of decline: (i) the chytrid was already present and widespread along the peninsula well before the first evidence of the decline of *B. pachypus*, and (ii) the geographic distribution of the declines does not match that of the chytrid (in historical or contemporary samples).

In suitable habitats along the Apennine chain, *B. pachypus* was considered as a common species until the mid 1990’s, when population declines and disappearances begun to be reported [38], [39]. Nevertheless, our results date back the presence of the pathogen to at least 1978. According to the ‘novel pathogen hypothesis’, the chytrid would undergo wave-like spreads, causing mass mortality of ‘naïve’ susceptible species as it arrives [5], [13], [21]–[23]. If this was the case, we should have observed waves of mortality long before the mid 1990’s, particularly when considering the estimated rates of spread of *B. dendrobatidis* (e.g. [21] but see also [24]). On the contrary, a time lag of more than 15 years separates the first evidence of chytrid occurrence from the observed declines. On the basis of our data, we cannot speculate about how and when the chytrid became established in the area (i.e. if it is endemic or if it was introduced once or many times, owing to anthropogenic transport or via natural long-distance dispersal). Nevertheless, these data allow us to exclude wave-like mortality of *B. pachypus* soon after chytrid arrival. Therefore, regardless of whether *B. dendrobatidis* is native or exotic to the region and when it arrived, it has not acted as a ‘lone killer’ (see also [46], [47]).

The spatial mismatch between chytrid occurrence and the decline of *B. pachypus* populations provides further evidence against the hypothesis that *B. dendrobatidis* acts as a ‘lone killer’. Indeed, the decline is affecting *B. pachypus* populations from the northern and central regions of its range, while no evidence of decline has been reported for the Calabria region. Nevertheless, our data showed that *B. dendrobatidis* is present in Calabria as well, and it was already there in the late ‘70s, indicating that southern populations are coping with chytrid infections for at least 35 years, without detectable signs of dying-offs or negative demographic trends [38], [39].

On the whole, the observations above have at least two important implications concerning the disease dynamics: 1) *B. dendrobatidis* did not cause extirpation of *B. pachypus* populations immediately after its arrival, but affected them during outbreaks in areas where it had previously become established, probably under the influence of other factors and 2) these factors are affecting *B. pachypus* populations differently along the species range. Noteworthy, spatial and temporal patterns were not only observed at the level of *B. pachypus* population declines, but also of *B. dendrobatidis* prevalence, although the latter should be taken with some caution owing to the higher probability of false negatives in historical samples.

Several factors have been proposed as ‘cooperating’ with *B. dendrobatidis* in promoting the decline of amphibians (reviewed in [18], [19], [48]–[50]). A role for factors that have a local action, e.g. changes in land-use or environmental contaminants, cannot be excluded with regard to the decline of single *B. pachypus* populations. Nevertheless, they are unlikely to explain the spatial and temporal pattern of declines, as they are managed at a local scale and not suitable to account for the extent of the observed process.

Although both field and laboratory experiments will be needed to reliably assess the nature of those factors interacting with *B. dendrobatidis* in priming the observed declines, we suggest that two of such factors could be the genetic diversity of host populations, that could account for the spatial pattern, and the recent climate change, that could account for the temporal pattern of declines.

The spatial pattern of the decline of *B. pachypus* is strikingly paralleled by the range-wide pattern of population genetic diversity. In a recent survey of the genetic variation among *B. pachypus* populations [51], the Calabria region was identified as the hotspot of intraspecific genetic diversity and the glacial refugium for this species, from where northern areas were re-colonized since the end of the last glaciation. Northern populations, although slightly differentiated from the southern ones, appeared genetically invariable, as expected for ‘recently’ founded populations (see also [52]–[54] and references therein). Populations with lower levels of genetic diversity have a higher probability of becoming genetically inbred, with potential consequences of lowered fitness and increased probability of facing higher extinction risks [55]–[57]. Furthermore, it is well established that low levels of genetic variation can be accompanied by higher susceptibility to emerging pathogens [55], [58]–[60]. In the present case, the pattern of population genetic diversity across the species range would comfortably accommodate the spatial pattern of declines, suggesting higher susceptibility to *B. dendrobatidis* of northern populations owing to their reduced adaptive potential (see also [61]).

Nonetheless, a hypothesis of a interaction between *B. dendrobatidis* and the reduced genetic diversity of northern populations in promoting population declines would left the temporal framework unexplained (i.e. the time laps since the first evidence of chytrid occurrence in the area and the observed declines). Among the factors that could help explaining such framework, climate is the only one that would act on a sufficiently large geographic scale, with marked and well-documented changes in recent years and an increasingly apparent impact on living systems [18], [19]. Along the Italian peninsula, mean temperatures have raised in the last decades, intensity and duration of heat-waves have increased, and the number of wet days has decreased [62], [63]. Changes in temperature regimes and precipitation patterns, as well as extreme climatic events, could have altered disease dynamics in the last decades, promoting disease outbreaks, as already suggested both for other chytrid-driven amphibian declines [29], [30]–[32] and for a wide range of distinct host-pathogen systems (e.g. [64]).

### Concluding Remarks

The assessment of the spatial and temporal distribution of emerging pathogens is of utmost importance in epidemiological research. Indeed, such an approach is providing increasingly useful insights into host-pathogen-environment relationships and disease dynamics.

In this work, analysis of the spatial and temporal distributions of *B. dendrobatidis* among samples spanning the whole Italian peninsula allowed us to exclude *B. dendrobatidis* as the ‘lone killer’ of the endangered species *B. pachypus*, as a scenario of wave-like mortalities soon after *B. dendrobatidis* arrival does not fit the spatial and temporal occurrence data gathered. Moreover, the temporal mismatch between the occurrence of *B. dendrobatidis* and the decline of *B. pachypus*, as well as different demographic trends of its southern vs. northern populations, indicate that other factors interact with *B. dendrobatidis* causing the observed declines.

Among the potentially interacting factors, we indicated two as the most plausible ones: 1) genetic diversity of host populations, which could have formed the basis for the spatial component of the observed declines, and 2) recent climate changes, which could have triggered changes in the outcome of host-pathogen interactions.

Both field and laboratory experiments are ongoing to test this hypothesis. Indeed, once validated, it would have at least one major implication of both theoretical and applied importance: management programs aimed at the genetic rescue of northern *B. pachypus* populations using genetic resources from southern populations, could have the potential to revert the observed negative demographic trend.
